# A new electrochromic copolymer composed of 4,7-di(thiophen-2-yl)benzo[c] [1,2,5]thiadiazole and 3,4-ethylenedioxythiophene

**DOI:** 10.55730/1300-0527.3456

**Published:** 2022-05-20

**Authors:** Kübra ÇELİK, Arif KIVRAK, Buket BEZGİN ÇARBAŞ

**Affiliations:** 1 Department of Energy Systems Engineering, Karamanoğlu Mehmetbey University, Karaman, Turkey; 2 Conductive Polymers and Energy Applications Laboratory, Karamanoğlu Mehmetbey University, Karaman, Turkey; 3 Department of Chemistry, Faculty of Sciences, Eskişehir Osmangazi University, Eskişehir, Turkey

**Keywords:** Conducting polymers, benzothiadiazole, electrochemical polymerization, electrochromic copolymer

## Abstract

A new electrochromic copolymer based on 4,7-di(thiophen-2-yl)benzo[c] [1,2,5]thiadiazole and 3,4-ethylenedioxythiophene was successfully obtained via the electrochemical polymerization method in the medium of electrolyte solution, which consists of 0.1 M tetrabutylammonium hexafluorophosphate and dichloromethane. In order to compare the electrochemical and chemical properties of the copolymer, the benzothiodiazole derivative and 3,4-ethylenedioxythiophene (EDOT) monomers, which are parts of the copolymer, are electrochemically polymerized, separately in the same environment and concentration with the copolymer. Poly(4,7-di(thiophen-2-yl)benzo[c][1,2,5]thiadiazole) (P(TBT)) and poly(3,4-ethylenedioxythiophene) (PEDOT) polymers were obtained. Before starting the electrochemical synthesis, the initial oxidation potentials of the monomers that form the copolymer were compared via the cyclic voltammeter method in this solvent electrolyte medium. Then, electrochemical and spectroelectrochemical characterizations of electrochemically synthesized polymers and copolymers were performed. New copolymer shows metallic blue and centaury blue in its neutral and oxidized states, respectively with a low band gap of 1.32 eV. Moreover, the copolymer has a 59% optical contrast and high coloration efficiency (324 cm^2^C^−1^) at 585 nm with a switching time of 2.2 s.

## 1. Introduction

Conductive polymers are capable of meeting the needs of many different applications, such as sensors [1–4], solar cells [5], light-emitting devices [6], capacitors [7], and electrochromic devices [8–13], with a creative design of chemical structures. Conjugated conducting polymers have a vital role in the fields of material science and energy because of their exceptional and tuneable properties (i.e. stability, conductivity, electronic and optical behaviors, etc.). While the properties of the monomer structure for the application are important in gold value, sometimes adding the functional group to the monomer structure may not be enough to obtain the expected electronic and optical properties of polymers. It is an undeniable fact that the effect of electrochemical and optical properties on the polymer main chain is higher than when a functional group is attached to the chemical structure of the polymer [14]. Under these conditions, most researchers have spent a lot of time on new monomers and their polymer/copolymer structures.

Recently, great attention has been given to the synthesis of low band gap polymers. Latest studies show that donor and acceptor units reduce the band gaps and thus facilitate the copolymerization of aromatic and quinoid heterocyclic structures [15]. In donor-acceptor-donor type polymers, benzo[c][1,2,5]thiodiazole, which is one of the strong acceptor chemical structures, and the structures used by adding a donor group with this structure (thiophene, furan, 3,4-ethylenedioxithiophene etc.) are generally used and low band gap polymers are obtained [16–18]. The electropolymerized polymers of 4,7-di-2-thienyl-2,1,3-benzothiadiazole have been popular due to their low band gap and rich optical and electrical properties [19,20]. The chemical structure modification with copolymerization method combines the electrochemical and optical properties of two units under the same roof. These properties are tuned and enriched by playing with the ratio of monomer feed ratios.

In this study, in the light of this information, it is hoped that the polymer obtained as a result of copolymerization of a comonomer structure consisting of thiophene and benzo[c][1,2,5]thiodiazole with the 3,4-ethylenedioxythiophene donor group, which has the higher electron-donating ability, will carry a lower band gap. For this purpose, a new and original electrochromic copolymer, which consist of 4,7-di(thiophen-2-yl)benzo[c][1,2,5]thiodiazole (TBT) and 3,4-ethylenedioxythiophene comonomers, will be electrochemically synthesized. Homopolymers PEDOT and P(TBT) will be synthesized in the same conditions with copolymer for comparison reasons. The obtained data will be compared with each other and the electrochromic properties of polymers will be discussed separately.

## 2. Materials and methods

All chemicals used were purchased from Sigma Aldrich without further purification. The 4,7-di(thiophen-2-yl)benzo[c][1,2,5]thiodiazole (TBT) comonomer was made according to reference [21]. Copolymerization was carried out using 3,4-ethylenedioxythiophene (EDOT) and TBT at a concentration of 10 mM each. Dichloromethane (DCM) and 0.1 M tetrabutylammonium hexafluorophosphate (TBAPF_6_) were used as electrolyte and solvent, respectively, during the electropolymerization process. During electrochemical polymerization and characterization of polymers, Pt disc (0.02 cm^2^) and Pt wire were used as working and counter electrodes, and Ag/AgCl electrode as reference electrode, respectively. While performing spectroelectrochemical studies, indium-tin oxide (ITO, Delta, Tech. 8–12 Ω, 0.7 cm × 5 cm) electrode was used as the working electrode. An Ivium Compactstat device was used as a potentiostat for electroanalytical studies. This device was also used simultaneously with the UV-vis spectrometer for spectroelectrochemical studies. FTIR spectra were recorded on Bruker Equinox 55 with an attenuated total reflectance (ATR).

## 3. Results

### 3.1. Electrochemical polymer and copolymer syntheses

**Figure 1:**
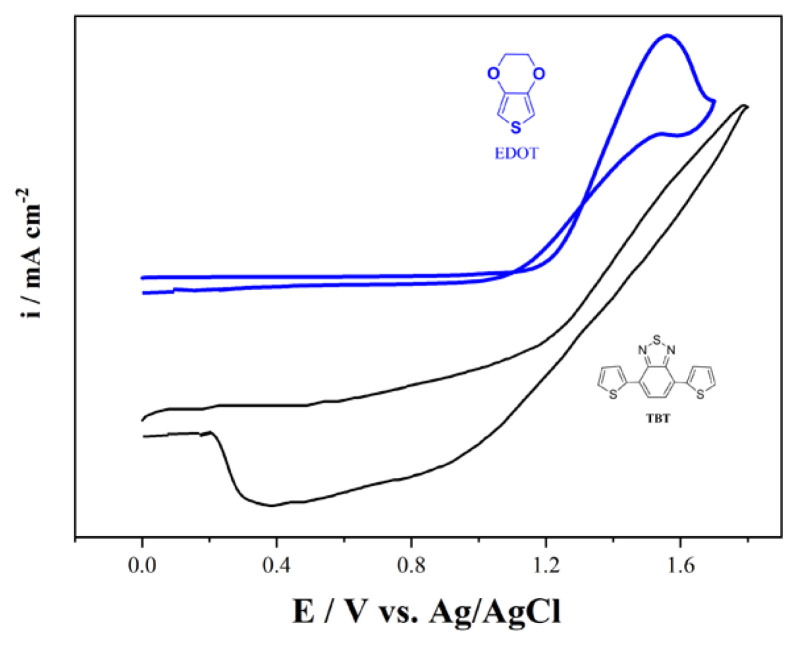
Oxidation potential comparisons of TBT and EDOT on Pt disc electrodes in the medium of 0.1 M TBAPF_6_/DCM solution with a scan rate of 100 mV s^−1^ vs. Ag/AgCl.

It is important to determine an appropriate oxidation potential when two different monomer structures become copolymers. For this purpose, the initial oxidation potentials of TBT and EDOT comonomer and monomer structures were compared by preparing 10 mM in 0.1 M TBAPF_6_/DCM medium under the same conditions (Figure 1). The TBT comonomer was first oxidized at around 1.5 V and the EDOT monomer at 1.55 V. The obtained results show that the oxidation potential difference between the two structures is quite low and sufficient for copolymerization.

**Figure 2:**
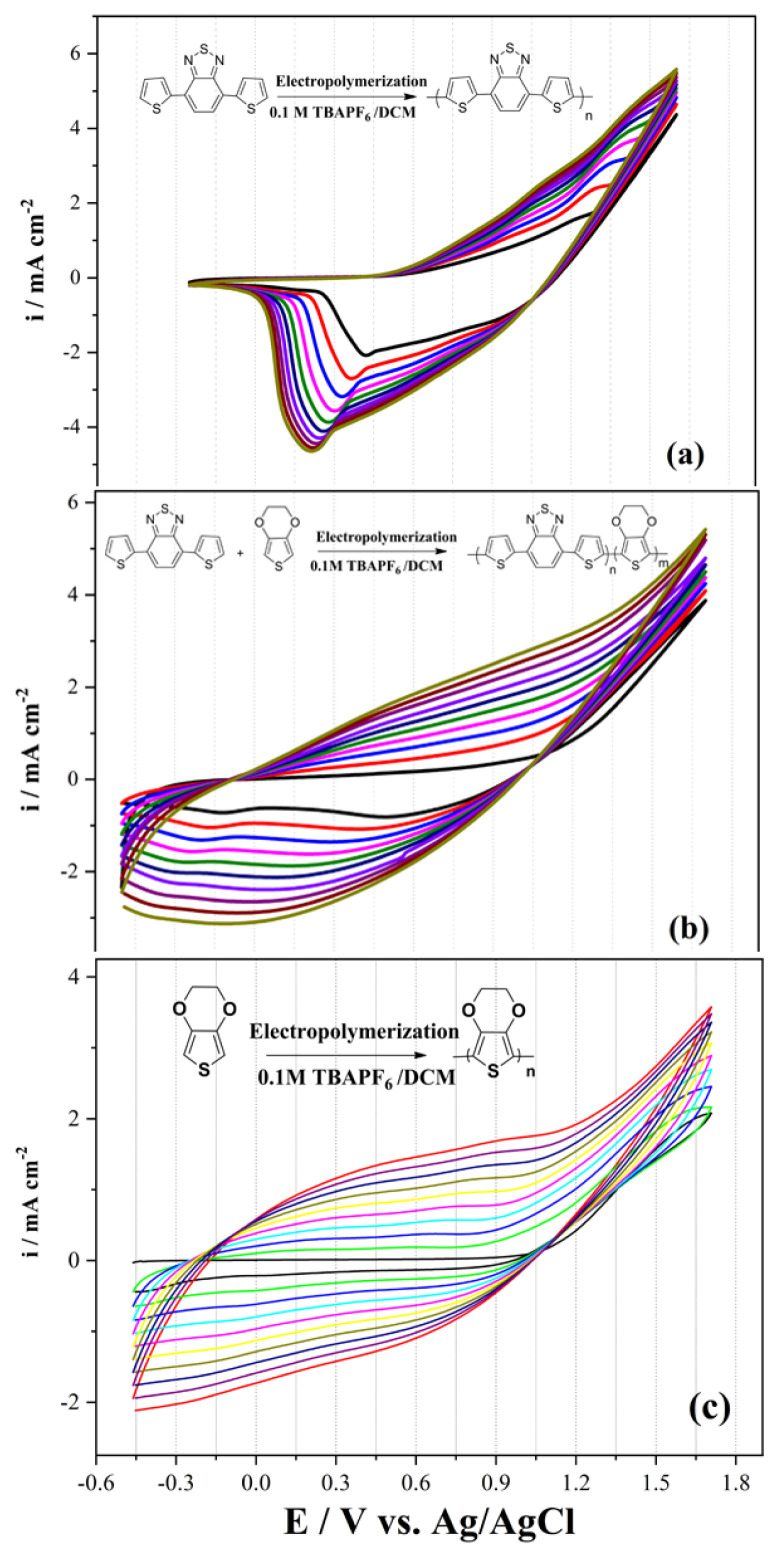
Electropolymerization studies of (a) TBT (b) copolymer (TBT:EDOT = 3:1) (c) EDOT solution with a scan rate of 100 mV s^−1^ vs. Ag/AgCl in 0.1 M TBAPF_6_/DCM solution.

The second process is to obtain the polymers and copolymers for comparison of their electrochemical and optical properties. All polymerization and copolymerization processes were carried out with the cycle voltammetry (CV) method in 10 cycles. Homopolymers; poly(4,7-di(thiophen-2-yl)benzo[c][1,2,5]thiodiazole) (P(TBT)), the copolymer (with a feed ratio of 3:1 (TBT:EDOT)) and poly(3,4 ethylenedioxythiophene) (PEDOT) were successfully synthesized in Figures 2a, 2b, and 2c, respectively.

**Figure 3:**
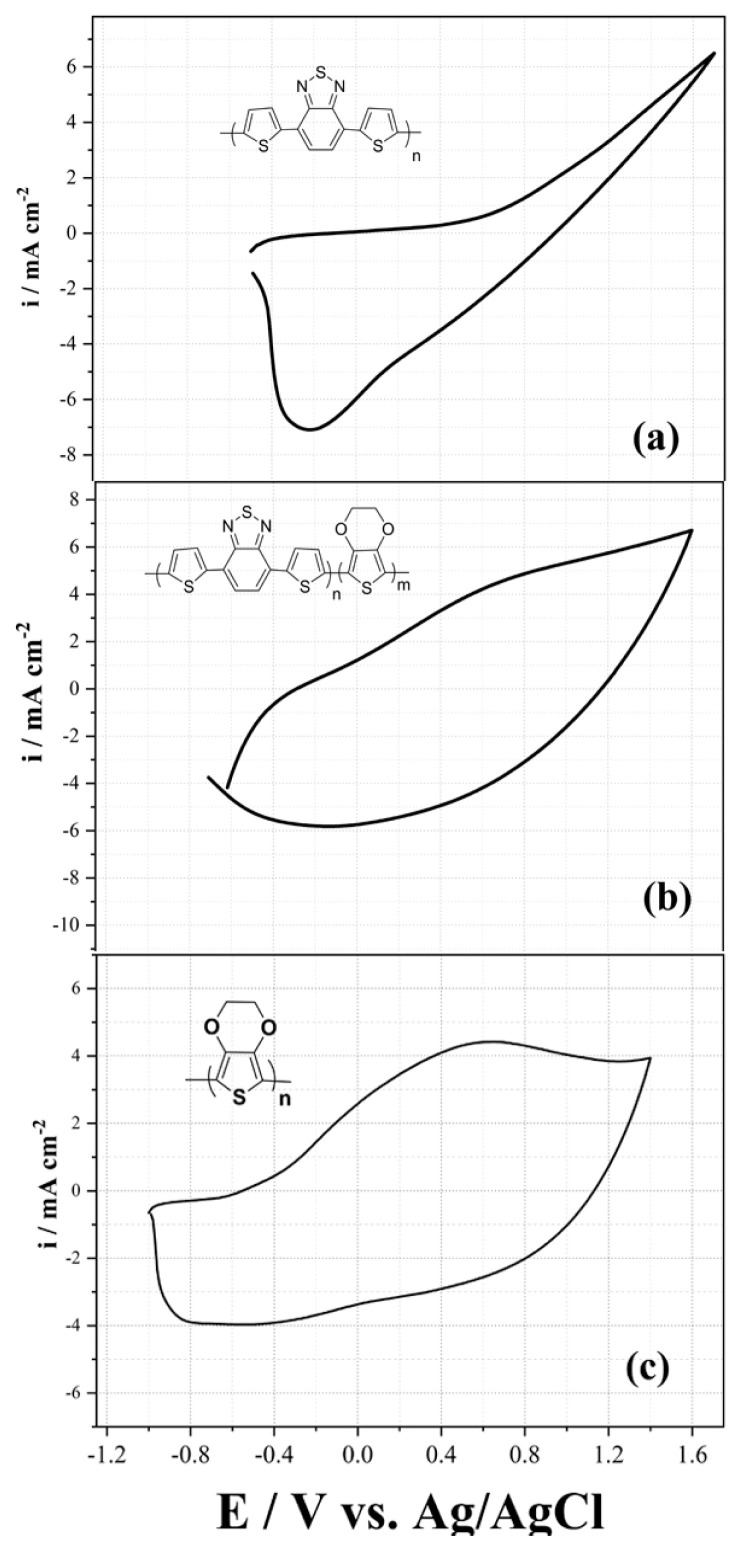
Electrochemical characterization behaviour of (a) P(TBT) (b) copolymer and (c) PEDOT polymers with a scan rate of 200 mV s^−1^ vs. Ag/AgCl in monomer free solution.

Then, the electrochemical behaviors of all polymers coated on the Pt disc were characterized in monomer-free medium by the CV technique, as seen in Figure 3. Before this process, electrochemically polymerized polymers were coated on the working electrode in a monomer medium, and then removed from this medium and cleaned in a monomer-free solvent medium to remove unpolymerized monomers and oligomeric structures. According to the data obtained, it was observed that the doping, dedoping values and capacitive CV curve properties of the obtained homopolymer and copolymer were different from each other. As seen in Figure 3a, homopolymers, P(TBT) has 1.2 V and −0.2 V doping and dedoping potentials respectively. For the copolymer, the doping and dedoping potential values were observed as 0.6 V and −0.2 V, respectively (Figure 3b). These values are 0.6 V (doping potential) and −0.6 V (dedoping potential) for the PEDOT homopolymer (Figure 3c).

**Figure 4:**
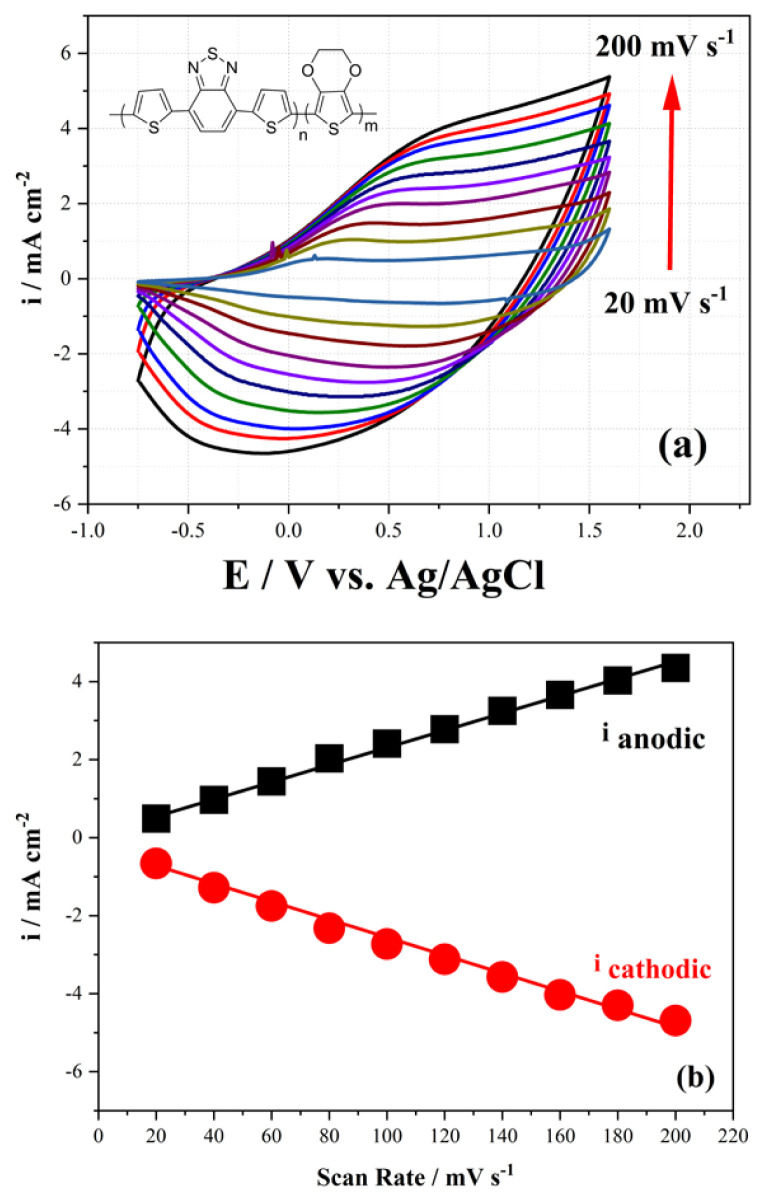
Electrochemical characterization behaviour of (a) copolymer with different scan rates between 20 mV s^−1^ and 200 mV s^−1^ with an increment of 20 mV. (b) Relationship of anodic (i_anodic_) and cathodic (i_cathodic_) current peaks as a function of scan rate.

In order to analyze the kinetic properties of the obtained copolymer, the copolymer film coated on the Pt disc surface was analyzed in monomer-free medium scanning with 20 mV s^−1^ a speed increment between the scan rates of 20 mV s^−1^ and 200 mV s^−1^. It has been observed that the anodic and cathodic current peak intensities of the copolymer increase and decrease linearly as the scanning speed increases, respectively (Figure 4). This showed that the kinetic behavior of the copolymer was not diffusional [22].

### 3.2. Electrochromic properties of copolymer

**Figure 5:**
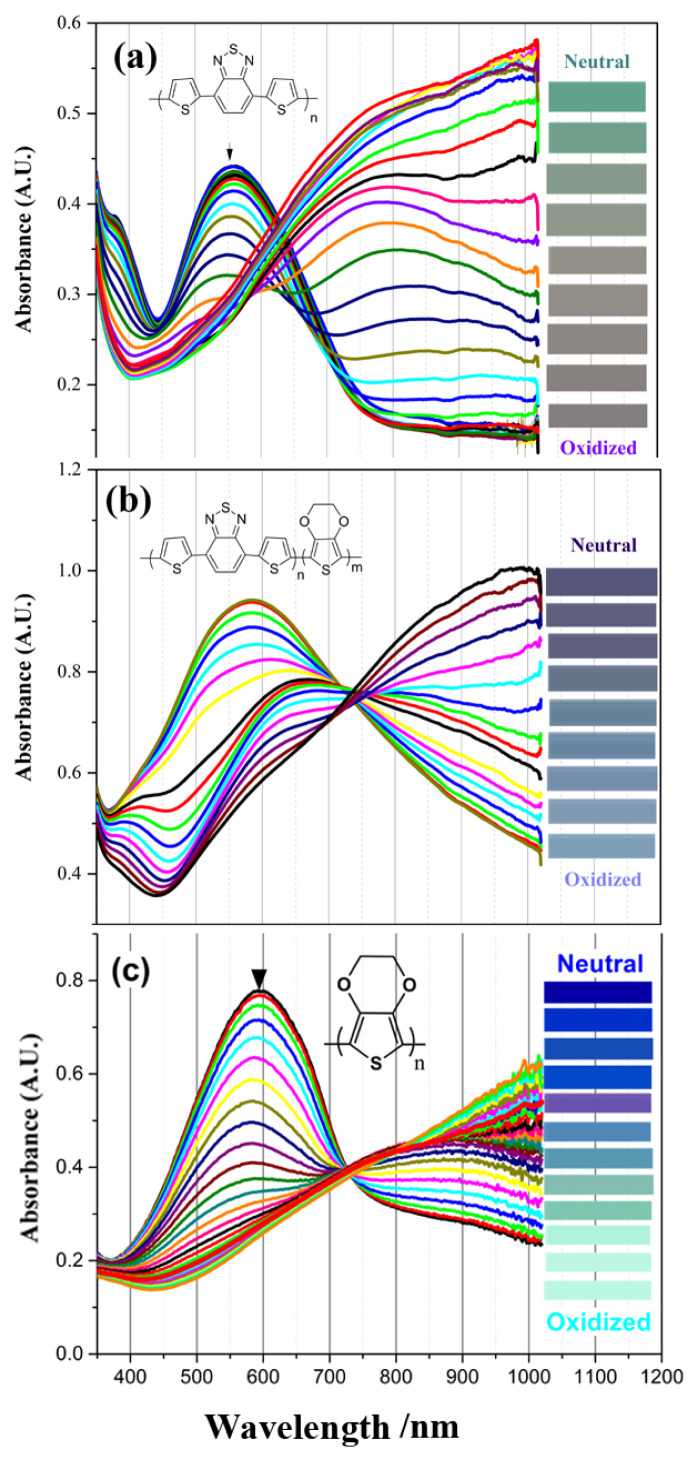
Spectroelectrochemical studies of (a) copolymer (c) PEDOT and inset figures; the colors of the polymers at their neutral and oxidized states in 0.1 M TBAPF_6_/DCM solution. The colors of the polymer film were put on each figure according to the L, a and b values of the polymer film during oxidation.

Electro-optical properties of polymer (P(TBT), PEDOT) and copolymer films were obtained by applying the necessary potentials for the oxidation of these materials and by monitoring the electronic absorption spectra. The mentioned UV-vis of polymers spectra are given in Figure 5. The maximum values of the optical absorption bands (π-π*) in the neutral states of P(TBT), copolymer and PEDOT appeared at 560 nm, 600 nm, and 585 nm, respectively (Figures 5a, 5b, and 5c). The isosbestic point of copolymer and PEDOT are located at around 760 nm and 725 nm, respectively. These values are assigned to the evolution of polaron band. It has been observed that with the application of oxidation potential to polymers and copolymer films, the absorption band intensities of π-π* transition bands for each polymer and copolymer decrease and new increasing bands are formed at higher wavelengths with the applied potential value. These newly formed bands are known as charge carriers, namely, polaron (Figures 5a, 5b, and 5c). The effect of both PEDOT and P(TBT) polymers on the formation of the absorption bands of the film for the copolymer was also observed.

The optical band values (E_g_) of the films were calculated from the beginning of the low energy value of the π-π * band transitions. Accordingly, E_g_ values were found to be 1.55 eV for P(TBT) and 1.60 eV for PEDOT, while copolymer has a lower band gap value of 1.32 eV. In Table, spectroelectrochemical data are given and compared for all three structures. It was also compared with the data of poly(4,7-di(2,3-dihydro-thieno[3,4-b][1,4]dioxin-5-yl)benzo[1,2,5]thiadiazole) (PBDT), which is a similar structure derivative of benzotriazole with EDOT [20]. When we compare the band gaps of the materials, it is observed that the band gap of the EDOT derivative PBDT is at the lowest value. It is obvious that the introduction of EDOT unit into the copolymer reduces the band gap [24–26].

The applied oxidation potential of the polymers and copolymer films caused the polymer films to have color changes, that is, to show electrochromic properties. Accordingly, polymers P(TBT) and PEDOT and copolymer films show cyan, dull blue and metallic blue colors in a neutral state, respectively, while in an oxidized state they show pale gray (P(TBT)), St. centaury blue (copolymer) and light blue (PEDOT) colors (see color photos in Table).

For an electrochromic polymer, especially when evaluating the suitability of the material for electrochromic applications, the important parameters are to analyze and present the switching time, optical contrast ratio and coloration efficiency (CE). In order to reveal these data, data were obtained by running the kinetic mode of the UV-vis spectrometer and the transition program between the neutral and oxidation potential values of the polymers and copolymer films in parallel at 10 s intervals in the potentiostat galvanostat device. All kinetic results are given in Table and calculated considering that the human eye is the most sensitive and at 95% of full contrast. As seen in Table, the optical contrast ratio of the P(TBT) and PEDOT structures were found to be 17% and 56%, respectively, while this value was calculated as 59% for the copolymer. This value of PBDT which is found in the literature is 23%. CE values were found according to following equation by using optical density ΔOD and injected/ejected charge density (Q_d_) during a redox step [26]. CE of copolymer was measured as 324 cm^2^C^−1^ (at 585 nm) which is a little bit lower than that of PEDOT (380 cm^2^C^−1^) and higher than that of P(TBT) and PBDT. Switching time (t_switching_) values are given at corresponding wavelengths, which are given in Table. The coloration and bleaching switching times at 585 nm are found as 2.2 s and 2.5 s, respectively. When the switching times are compared, the lowest switching time belongs to PBDT and the t_switching_ values are between that of PEDOT and P(TBT).

### 3.3. Electrochemical and spectroelectrochemical stability tests of copolymer

**Figure 6:**
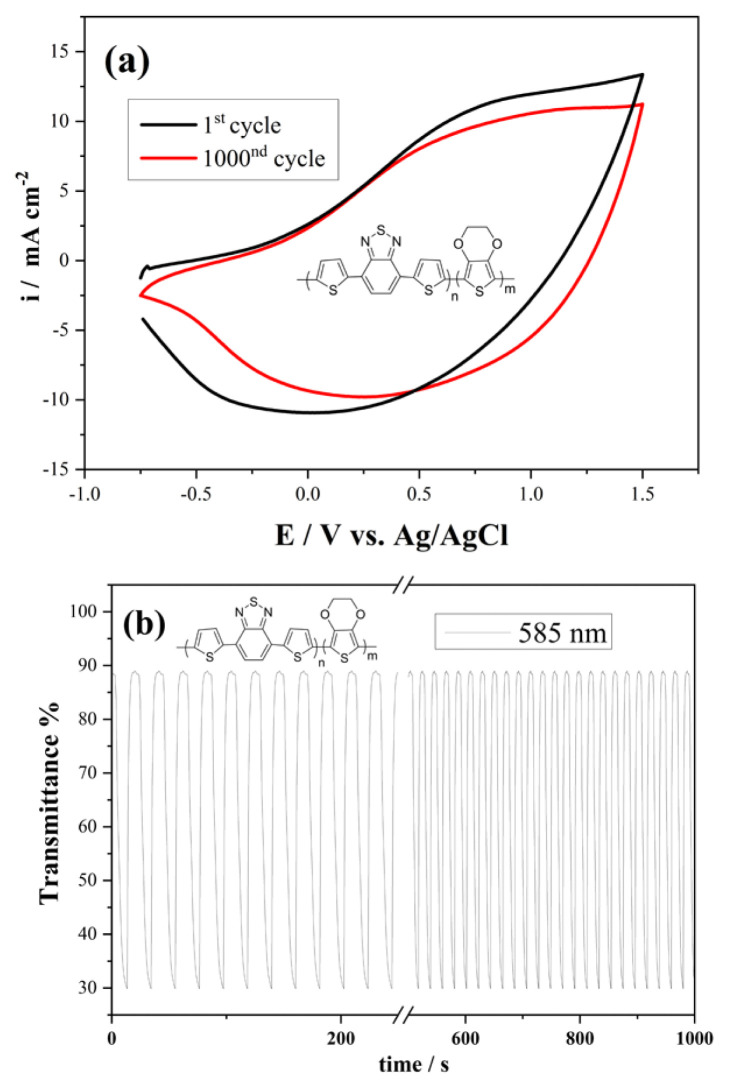
(a) Electrochemical and (b)spectroelectrochemical (at 585 nm switched for 10s between their neutral and oxidized states) stabilityexperiments for copolymer.

The durability of polymers during switching and cyclic behavior is very important in device applications such as capacitors, batteries and electrochromic devices. For this purpose, the electrochemical stabilities of the copolymer coated on the Pt disc were determined by CV technique in a monomer-free environment and under N_2_ gas. According to the results obtained, as seen in Figure 6a, the copolymer retains approximately 75% of its electrochemical activity after 1000 cycles.

Spectroelectrochemical stability tests were performed with the kinetic mode of the UV-vis spectrometer working simultaneously with the potentiostat-galvanostat device (Figure 6b). Data were obtained for a time period of 1000 s while the copolymer was switched between neutral and oxidation potentials at 585 nm. According to the data obtained, the copolymer lost only 5% of its optical activity during the 1000 s period. These results show that the copolymer can be used as a potential material with good stability in technological applications such as energy storage and electrochromic devices.

### 3.4. Chemical structure analysis of copolymer

The chemical structure analysis of copolymer formation was proved by using the FTIR spectrum. As seen in Figure S1, the spectra of monomers (TBT and EDOT) and copolymer were drawn by superposing each data in the same spectra (Figure S1). The results show that the spectra of copolymer were very broad and only some peaks were analyzed. After electrochemical copolymerization of TBT with EDOT, the disappearance of peaks at 773 cm^−1^ and 3100 cm^−1^ are evidence of the polymerization of 2.5 positions of the thiophene ring. In addition, the new sharp peak that appears at 840 cm^−1^, is attributed to the PF_6_^−^ counter anion.

## 4. Conclusion

In this study, a new copolymer with the chemical structures of thienyl benzothiadiazole and EDOT was electrochemically polymerized. For this purpose, the polymers ( P(TBT) and PEDOT) were also synthesized under the same conditions as the copolymer. Electrochemical and spectroelectrochemical data show that the copolymer was successfully synthesized. The band gap was found to be 1.32 eV for the copolymer and the copolymer shows an electrochromic property. It was found that the color of copolymer turned to metallic blue and centaury blue in the fully reduced and oxidized states, respectively. While the optical contrast ratio of the copolymer (59% at 585 nm) was found to be higher than that of the P(TBT) and PEDOT polymers, the coloration efficiency was found as 324 cm^2^C^−1^ with a switching time of 2.2 s. Electrochemical (for 1000 cycled polymer) and spectroelectrochemical (for polymer switched for 1000 s) stability tests showed that the copolymer was stable. This study shows that the copolymer has the potential to be used in electrochromic devices and energy-saving smart glass applications.

## Supplementary Information

Figure S1. FTIR spectra for (a) TBT (b) EDOT, and (c) copolymer.

## Figures and Tables

**Table:** Spectroelectrochemical properties comparisons of copolymer and other similar chemical structures.

Chemical structures	λ_max_ (nm)	% ΔT	t_switch_ (s)	E_g_^opt^ (eV)	CE (cm^2^/C)	Colors	
						Neutral	Oxidized
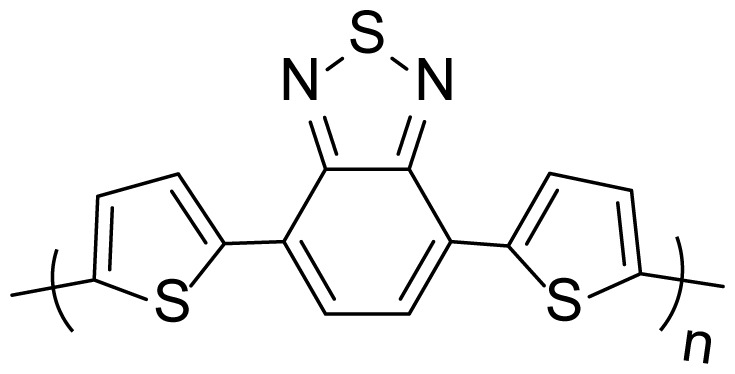	560^*^	17 18^*^	t^c^: 2.5 t^b^: 4.0	1.55 1.50^*^	105	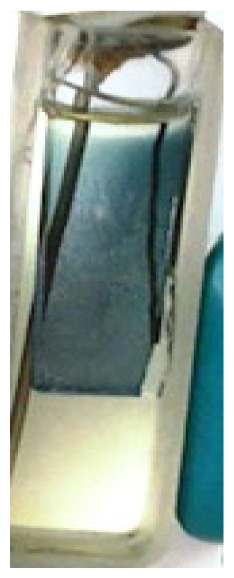 cyan	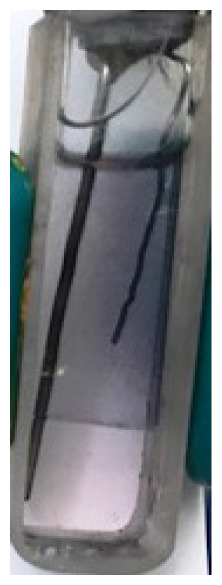 pale gray
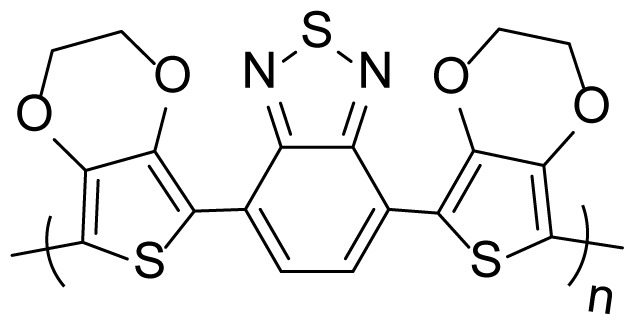	755^**^	23^**^	0.4^**^	1.19^**^	130^**^	green^**^	transparent blue^**^
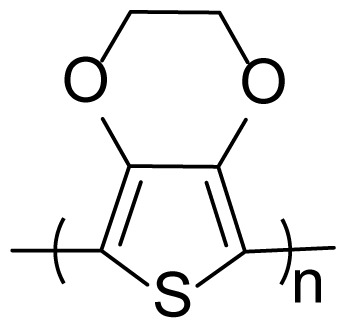	600^***^	56^***^	t^c^: 0.8 t^b^: 1.5	1.6^***^	380^***^	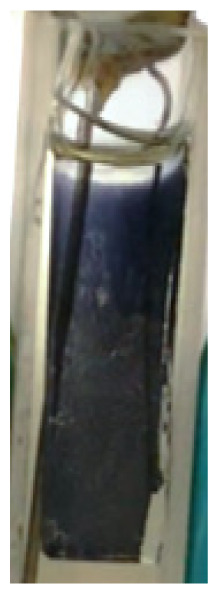 black blue	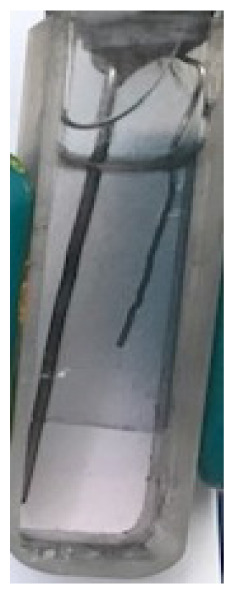 transparent blue
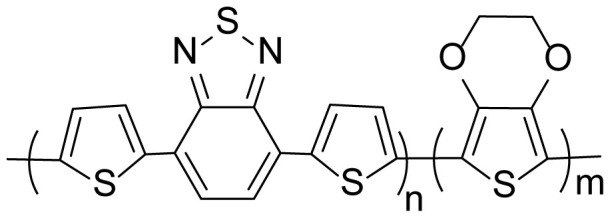	585	59	t^c^: 2.2 t^b^: 2.5	1.32	324	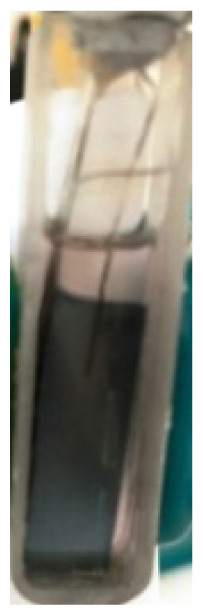 metalic blue	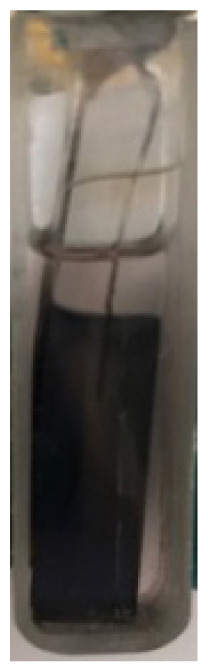 centaury blue

(^*^[16],[24]; ^**^[23]; ^***^ [25]) t^c^ (coloring time), t^b^ (bleaching time). The calculations were done at 95% of full contrast. Optical Density (ΔOD) = T_colored_/T_bleached_, where T _colored_ maximum transmittance value at oxidized state and T_bleached_ maximum transmittance value at neutral state. CE = ΔOD/Qd, where Qd is the charge density during switching cycle between the oxidized and neutral states [26]. Photographs of polymer films were taken during the experiment.
