# Supplementary figures and images for: A new electrochromic copolymer composed of 4,7-di(thiophen-2-yl)benzo[c] []thiadiazole and 3,4-ethylenedioxythiophene
Source: Turk J Chem. 2022 May 20;46(5):1516–23. doi: 10.55730/1300-0527.3456 (PMC10390108; doi:10.55730/1300-0527.3456)

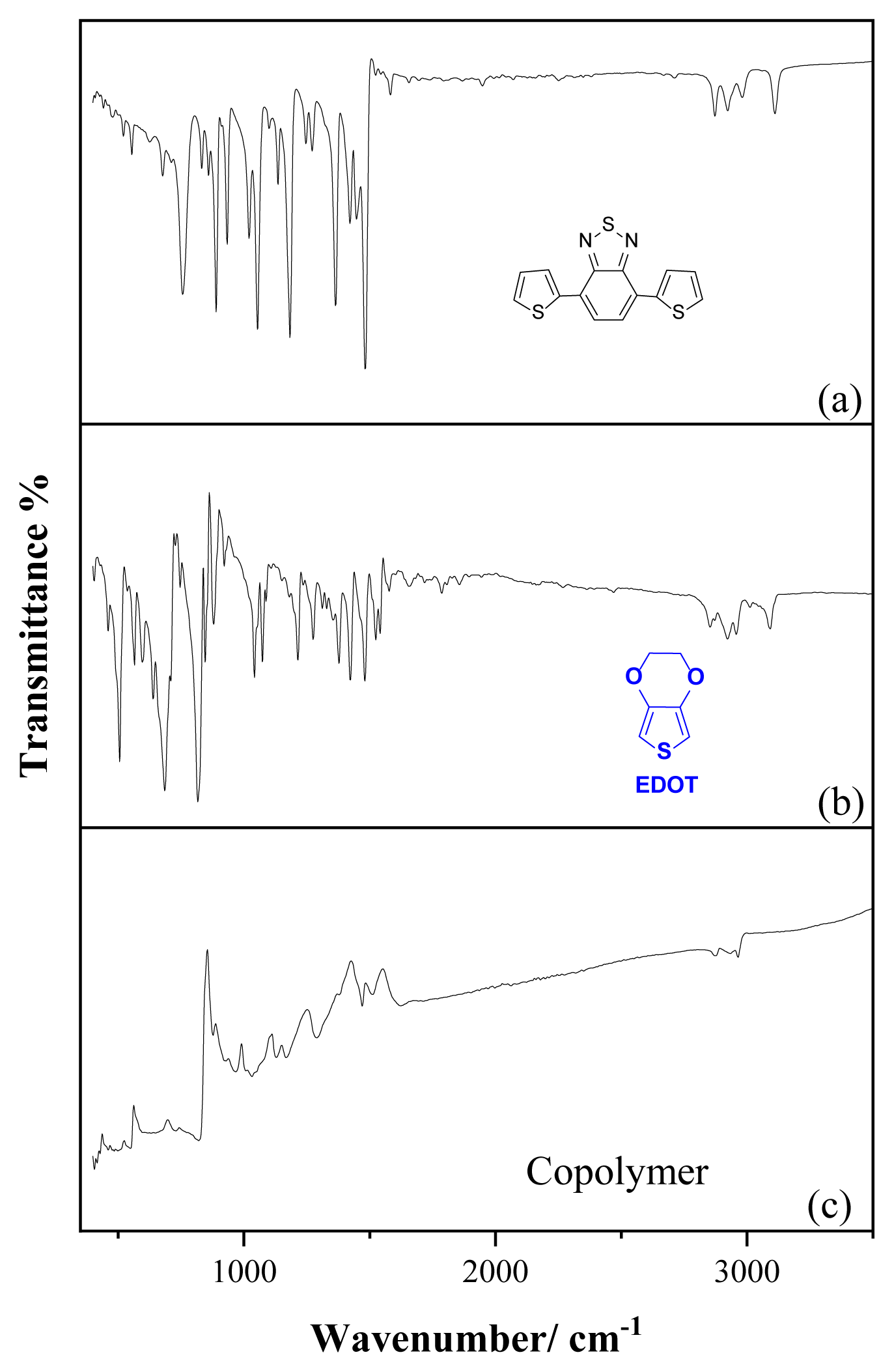

Supplement: Figure S1. — FTIR spectra for (a) TBT (b) EDOT, and (c) copolymer. [file turkjchem-46-5-1516s1.tif]
